# Work-Related Satisfaction among Clinicians Working at Inpatient Treatment Facilities for Substance Use Disorder: The Role of Recovery Orientation

**DOI:** 10.3390/ijerph18147423

**Published:** 2021-07-12

**Authors:** Dagny Adriaenssen Johannessen, Trond Nordfjærn, Amy Østertun Geirdal

**Affiliations:** 1 Blue Cross East, 0182 Oslo, Norway; 2 Department of Social Work, Child Welfare and Social Policy, OsloMet—Oslo Metropolitan University, 0130 Oslo, Norway; amyoge@oslomet.no; 3 Department of Psychology, Norwegian University of Science and Technology (NTNU), 7491 Trondheim, Norway; trond.nordfjarn@ntnu.no; 4 Department of Research and Development, Clinic of Substance Use and Addiction Medicine, St. Olavs University Hospital, 7006 Trondheim, Norway

**Keywords:** inpatient treatment, job satisfaction, psychosocial factors, Norwegian recovery self-assessment (RSA-N), regression analysis

## Abstract

Several psychosocial factors have been suggested as facilitators of change among inpatients treated for substance use disorder (SUD). Research suggests that staff members are also influenced by the practice in which they are involved, and by contextual psychosocial factors at their treatment facilities. This cross-sectional questionnaire survey study was conducted to investigate the role of recovery-orientated interventions in describing work-related satisfaction among clinicians at inpatient SUD treatment facilities. The respondents (*n* = 407) rated items indicating work-related satisfaction and the degree of recovery orientation at their treatment facilities. The main findings of two block regression analyses indicated that clinicians’ work-related satisfaction was positively influenced by inpatients’ opportunities to pursue their goals and choices, and negatively influenced by inpatient involvement. The change in clinicians’ work-related satisfaction could not be described by the degree of individually tailored and varied interventions at the treatment facility. Clinicians should be supported and involved in the process of implementing measures to increase inpatient involvement in the treatment programmes, and treatment measures that enable inpatients to pursue their goals and choices should be enhanced. The findings of this and previous studies indicate that a recovery-oriented framework promotes clinicians’ work-related satisfaction and has an enabling influence on both inpatients and clinicians.

## 1. Introduction

Work-related satisfaction is described as a positive emotional state emerging from appreciation associated with work, which may affect employees’ behaviour and performance at work [1,2]. Factors such as mastery of work and expectation fulfilment contribute to better work-related satisfaction, and mastery of work may also contribute to enhanced quality of life (QoL) and work engagement. Furthermore, acquiring useful qualifications to overcome challenges at work has been associated with better work engagement and greater work-related satisfaction [3,4].

Clinicians working in treatment for substance use disorder (SUD) report an overall high level of burnout and low work-related satisfaction [5,6]. In this regard, high workload, burnout and work-related stress have been associated with low work-related satisfaction and QoL among clinical staff members [4,7,8,9]. The differences in work-related satisfaction have not been attributed to gender [6,10], profession [6,11], or seniority [12] among clinicians working in SUD services. While some studies show that work-related satisfaction increases with age [12], others have found no such association [6,10]. The severity of dependency is normally decisive for service intensity, where people with severe SUD are provided with high-intensity services as compared to low-intensity services [13]. The findings from previous research on the association between work-related satisfaction and service intensity are diverging. Some suggest that clinicians who work in low-intensity services for people with SUD or mental health problems, report higher work-related satisfaction than clinicians in high-intensity services [14], while others find no differences in work-related satisfaction among clinicians in low- versus high-intensity services [6,8].

As Broome, Knight, Edwards and Flynn [8] argued, the interest in employees’ work-related satisfaction can partly be attributed to its ability to influence clinicians’ performance at work, as well as the service quality. For example, previous research showed that low work-related satisfaction among clinicians was associated with negative attitudes towards inpatients [15,16], and diminished service quality [17,18]. Factors such as high workload [8], rapid turnover [19,20], and low social support among the employees [5,21,22] have been associated with lower work-related satisfaction in SUD and mental health services. Additionally, conflicting expectations between inpatient and employers may burden healthcare clinicians and influence their work-related satisfaction adversely [23].

### 1.1. Enabling Environments

People who need substantial change and development to manage their everyday lives and overcome the psychosocial consequences of SUD, often require specialised inpatient treatment [24,25,26,27]. People with SUD have long been the subject of controlling measures in society in general, and in SUD treatment in particular [28,29], and the environment at the services to which people refer to undergo vast change processes, such as inpatient SUD treatment, is tightly controlled [30]. The treatment practice and the service staff that undertake it perform an essential function in, and exert a profound influence on, inpatients’ everyday lives and their change processes [30].

Some of the core tasks at facilities providing inpatient SUD treatment include promoting change and development [30,31,32]. Environmental factors that have been shown to foster positive change (i.e., contextual psychosocial factors that enable development, satisfaction and well-being) include user involvement and empowerment, diversity of treatment options, individualised treatment measures, and the opportunity to pursue individually defined goals [33,34,35,36].

These factors represent some of the primary values in the notion of recovery [37,38,39], which is defined as a process of change in life domains that are affected by the negative psychosocial consequences of SUD or mental health problems [40]. Over the past few decades, a recovery orientation framework has increasingly been endorsed in services for people with SUD or mental health problems worldwide [41,42,43,44]. Services that draw values and measures from the recovery tradition, such as inpatient SUD treatment services, are known as recovery-oriented services [45,46]. Patients in recovery-oriented treatment report better treatment outcomes (e.g., self-efficacy, hope, supporting relations), compared to patients in services that are not recovery-oriented [47,48]. Additionally, patients rate the quality of recovery-oriented services more favourably than services that are not recovery-oriented [49]. In line with this, patients who are satisfied with the treatment service stay longer and experience better treatment outcomes, in terms of well-being, social function and substance use [50,51].

While several factors have been suggested as facilitators of change and development among inpatients, research suggests that staff members are also affected by contextual psychosocial factors that are emphasised in the therapeutic orientation at treatment facilities [11,12,52,53]. Studies have shown that recovery-oriented mental health clinicians experience greater work-related satisfaction than those who perceive the practice as less recovery-oriented [11,12,52]. As shown in previous research, recovery-oriented clinicians are more optimistic on behalf of the patients recovery opportunities [54,55], and experience better social support in the workforce [53]. Other therapeutic orientations that emphasise the importance of an enabling psychosocial environment are patient-centred and trauma-informed care. These therapeutic orientations share values that are similar to those of recovery orientation, and have also been associated with greater work-related satisfaction among mental health and SUD clinicians [56,57].

### 1.2. Aims of This Study

Low work-related satisfaction among staff members may reduce inpatients’ opportunities to pursue change and development [15,16,17,18,30]. Knowledge of the measures that concurrently contribute to greater work-related satisfaction among clinicians, and better conditions for inpatients to attain change and development, is arguably of interest for the SUD treatment field. This study contributes to the literature by exploring the association between environmental factors that enable inpatients to attain change and development, and factors that influences work-related satisfaction among staff members.

Based on the findings of previous research on clinicians working with SUD or mental illness, which have been reviewed during the introduction, we hypothesised that environmental psychosocial factors at SUD treatment facilities play an important role in clinicians’ work-related satisfaction. As there is a lack of knowledge due to the role of recovery-oriented interventions among clinicians in SUD, the study aimed to investigate the role of recovery-oriented interventions in describing clinicians’ perceptions of work-related satisfaction at inpatient SUD treatment facilities.

## 2. Materials and Methods

### 2.1. Participants and Procedure

This cross-sectional study involved clinical staff members working in SUD treatment facilities that provided long-term (≥six months) inpatient treatment for people with SUD in Norway. As in Europe and other western countries, SUD treatment in Norway emphasises a biopsychosocial understanding of SUD [58,59,60]. Specialised inpatient SUD treatment is therefore interdisciplinary, consisting of social, medical and psychological interventions and measures. Consequently, psychologists, social workers, nurses, medical doctors and psychiatrists are usually employed at such facilities.

Fifty-four facilities providing long-term inpatient SUD treatment were invited to participate in this study. Fifty facilities accepted the invitation and agreed to take part. The participating facilities employed 933 clinical staff and offered 991 beds for SUD inpatients.

A research coordinator assigned to each participating facility informed clinical staff members of the study’s aim and methods and provided them with a link to a questionnaire via e-mail. All staff members were provided with the same questionnaire, regardless of profession. The questionnaire comprised 40 items in total and took approximately 15 min to complete. Data collection took place from August to October 2020.

### 2.2. Measures

#### 2.2.1. Predictor: Recovery Orientation

The Norwegian version of the recovery self-assessment (RSA-N) was used to explore the degree to which clinicians working at inpatient SUD treatment facilities perceived their practice as recovery-oriented. The recovery self-assessment (RSA) [61] was developed in the United States (U.S) and has been extensively used to assess recovery orientation in mental health and SUD services see e.g., [11,12,62,63,64]. In a validity study of the RSA-N, the five-factor structure originally obtained by O’Connell, Tondora, Croog, Evans and Davidson [61] could not be replicated. Consequently, an alternative three-factor structure was suggested for RSA-N [65]. A confirmatory factor analysis of the three-factor solution showed acceptable approximate fit indices (RMSEA (90% CI) (0.059 (0.049–0.069), CFI (0.89)), and good internal consistency for the overall instrument (Cronbach’s *α* = 0.88) and its following three subscales: *goals and choice* (*α* = 0.82), *involvement* (*α* = 0.74) and *individually tailored and varied* (*α* = 0.75) [65].

The RSA-N consists of 23 items rated on a 5-point Likert scale (1 = ‘strongly disagree’ to 5 = ‘strongly agree’), with two additional response options (‘not applicable’ and ‘don’t know’) [65]. The two additional options were coded as system-missing values. The *goals and choice* subscale (nine items) collects information about the extent to which inpatients’ individually defined goals are promoted by the service staff and whether inpatients’ individual choices are respected. The *involvement* subscale (six items) gathers information on the degree to which inpatients are involved in the development of the treatment programme and whether they are involved in the planning of their own treatment. The third subscale, *individually tailored and varied* (eight items), is related to the degree to which the treatment facilities offer a diversity of treatment measures and whether the measures can be tailored to inpatients’ individual needs. The scores of the individual subscales provide information that can indicate potential areas of improvement for establishing a recovery-oriented environment at treatment facilities.

Following O’Connell, Tondora, Croog, Evans and Davidson [61], the total mean score of all 23 items and the total mean scores of the items in each subscale were calculated (ranging from 1 to 5). A high mean score indicates that clinicians perceive the practice as recovery-oriented, whereas a low score indicates the contrary.

#### 2.2.2. Outcome Variable: Work-Related Satisfaction

The General Nordic Questionnaire for Psychological and Social Factors at Work QPS_Nordic_ [4] measures psychological and social factors at work and has been validated for use in the Nordic countries [66]. In this study, the scales *positive challenges at work* and *mastery of work* were used. These scales are hypothesised to measure two factors associated with well-being and work-related satisfaction [3,4]. The *positive challenges at work* subscale comprises three items related to employees’ perceptions of the usefulness of their skills and knowledge, and of the work as meaningful and positively challenging. The *mastery of work* subscale consists of four items regarding employees’ evaluation of their effectiveness and work ability [66]. Each item is rated on a 5-point Likert scale ranging from 1 (‘very seldom or never’) to 5 (‘very often or always’). Following Skogstad [3], the total mean score of each subscale was obtained by computing the responses on each included item in the scale and dividing them by the number of items (ranging from 1 to 5). A high mean score indicates that clinicians perceive a high degree of mastery and positive challenges at work. In this sample, the alpha coefficients for the *positive challenges at work* and *mastery of work* subscales were acceptable (*α* = 0.75 and *α* = 0.72, respectively).

#### 2.2.3. Control Variables: Demographic Information

Seven items in the questionnaire collected information about the respondents’ personal and professional characteristics. Age, experience in the SUD field and experience in inpatient SUD treatment were measured in years (continuous variables). Items collecting information on gender, job title and whether the participants had patient contact included pre-specified response options as well as the option to give a written answer (categorical variables).

The *quantitative job demands* scale of QPS_Nordic_, which comprises four items rated on a 5-point Likert scale (1 = ‘very seldom or never’ to 5 = ‘very often or always’), was used to assess participants’ perceptions of workload and time pressure at work [66]. The scale showed good internal consistency (*α* = 0.81). As in the other scales, the total mean score was calculated (ranging from 1 to 5) [3]. A high mean score indicates that clinicians perceive high quantitative demands at work.

### 2.3. Ethics Approval

Along with the link to the questionnaire, clinical staff members received written information via e-mail. Participants gave their consent to take part in the study by responding affirmatively to the first item in the questionnaire, with the following: “I give my consent to participate in the study and to my answers being stored in Sensitive Data Services (TSD) and used for research purposes.” The study protocol was approved by the Norwegian Centre for Research Data (NSD; reference number: 883511) and followed the Declaration of Helsinki ethical principles.

### 2.4. Statistical Analysis

Multiple block regression analysis with ordinary least-square estimation was performed to investigate the role of clinicians’ personal and professional characteristics, and the role of recovery orientation in work-related satisfaction at inpatient SUD treatment facilities. Two separate multiple regression analyses were specified using the hypothesised outcome variables (*positive challenges at work* and *mastery of work*) separately. To investigate the role of recovery orientation in clinician’s reported work-related satisfaction, the three RSA-N subscales—*goals and choice*, *involvement* and *individually tailored and varied*—were included block-wise as predictors in the separate regression models. The *quantitative job demands* scale scores and the demographic characteristics were used as control variables.

No collinearity between the predictive variables was found as the variance inflation factors (VIF) were between 1.0 and 1.5. The Shapiro–Wilk test was used to explore the distribution of residuals of the outcome variables, with significant *p*-values indicating a non-normal distribution [67]. The Box-Cox transformation was used to normalise the distribution of residuals in outcome variables that yielded significant results in the Shapiro–Wilk test. All analyses were performed both with and without transformation. The results were similar in terms of significant contributions of the control variables and the hypothesised predictive variables, as were the results estimating the direction and strength of standardised regression coefficients (*β*). For the sake of simplifying the interpretation of both the statistical and substantial significance of the results, the untransformed outcome variables were used in the multiple regression analyses [68]. Using the transformed outcome variables, the Shapiro–Wilk test yielded *p*-values of 0.1 in both regression models, indicating an acceptable distribution of residuals. Using the untransformed outcome variables, the results of the Shapiro–Wilk test showed acceptable results for *mastery at work* (*p* = 0.10) and significant results for *positive challenges at work* (*p* < 0.01).

Cook’s distance scores were used to identify extreme observations with a high impact. High values indicate that the extreme observations have leverage. Values of ≤1 were considered acceptable [69]. All Cook’s distance scores in the data sample were less than 0.01.

A block-wise forward selection procedure was applied to specify the two separate regression models. First, the hypothesised predictive variables and the control variables were entered into the model. The variables were assessed based on their ability to predict significant variance in the outcome variables. Significantly predictive control variables were entered into the first block of the regression model. The hypothesised predictive variables were then entered one by one, ordered from highest to lowest impact, into the subsequent blocks. To simplify the models, control variables that did not contribute significantly to predicting clinicians’ scores on the outcome variable were not included in the main regression analyses [69,70]. Values of *p* < 0.05 were considered statistically significant.

Mean imputation, as described by Christophersen [71] (the item’s mean score plus the subscale’s mean score divided by two), was used on items with a minimum 90 percent or more valid responses [72].

Analysis of missing value patterns and imputation of system-missing values were performed using IBM SPSS Statistics version 27 (IBM, Armonk, NY, USA), while jamovi version 1.2.27 (jamovi, Sydney, Australia) was used for all other analyses [73].

Two items of the RSA-N had more than 10 percent system-missing values. Mean imputation was therefore not used for these two items. Moreover, one participant had more than 56 percent system-missing values on the overall RSA-N, and mean imputation was not used, as suggested by the developers of the original instrument [74].

## 3. Results

### 3.1. Sample Characteristics

Of the 933 invited clinical staff members from the 50 inpatient SUD treatment facilities, 426 returned the questionnaire (46 percent response rate). Among the respondents, 95.5 percent (*n* = 407) reported that they worked directly with the inpatients at their respective treatment facilities (i.e., clinically). The respondents who did not work clinically were excluded from the analyses.

Table 1 shows the participants’ personal and professional characteristics in numbers and percentages (categorical variables), and means ± standard deviations, due to the job demands scale (continuous variables). The participants’ mean age was around 45 years. In line with the gender distribution that was shown in previous research with clinicians in SUD or mental health services [8,11,12,22], almost two-thirds of the participants in this study sample were female. Nearly one out of five were social workers, and about one in three were medical staff. The least represented group was that of ‘psychologists or therapists’. The largest group was ‘other staff’, which included peer specialists, environmental therapists, financial counsellors, and job counsellors. The average score on the *quantitative job demands* scale was 2.9. The ‘psychologist or therapist’ group had the highest mean score, whereas the ‘other staff’ group had the lowest. The participants had a mean of almost ten years of experience in the SUD field, and about eight years’ experience specifically in inpatient SUD treatment.

### 3.2. Positive Challenges at Work

The preliminary analysis showed that among the control variables, age contributed significantly to predicting the clinicians’ scores on the *positive challenges at work* scale. The remaining control variables had no significant contribution, and were therefore not included in the main analysis (*p*-values ranged from 0.11 to 0.80).

Table 2 presents the results of the first multiple block regression analysis, with *positive challenges at work* as the hypothesised outcome. Age was included in the first block, followed by *goals and choice*, *involvement*, and *individually tailored and varied*. In total, these variables accounted for 20 percent of the variance in the *positive challenges at work* score (R^2^ = 0.20). Age accounted for 6 percent of the variance, suggesting that an older age was significantly associated with the perception of having positive challenges at work (*t* = 4.41, *p* < 0.001). *Goals and choice* accounted for 12 percent of the variance, and represented a unique significant contribution, indicating that clinicians who perceived that the treatment facility promoted inpatients’ goals and choices were significantly more likely to report experiencing positive challenges at work (*t* = 6.44, *p* < 0.001). *Involvement* showed a unique significant contribution to explain the variation in the clinicians’ scores on the *positive challenges at work* scale, indicating that higher levels of inpatient involvement at the treatment facility were associated with lower scores (*t* = −2.06, *p* < 0.05). Finally, the *individually tailored and varied* subscale had no significant contribution to variance in the clinicians’ scores (*t* = 0.33, *p* = 0.70).

### 3.3. Mastery of Work

The preliminary analysis showed that *quantitative job demands* contributed significantly to predicting clinicians’ *mastery of work* scores. Other control variables did not significantly affect clinicians’ reports of mastery of work, and were therefore not included in the main analysis (*p*-values ranged from 0.06 to 0.95).

*Quantitative job demands* was included in the first block of the second multiple regression analysis, followed by the hypothesised predictive variables (*goals and choice*, *involvement* and *individually tailored and varied*). In total, these variables accounted for 20 percent of the variance in the clinicians’ *mastery of work* scores (R^2^ = 0.20). As shown in Table 3, *quantitative job demands* contributed significantly, accounting for 5 percent of the variance (*t* = −3.95, *p* < 0.001). Clinicians who perceived high quantitative job demands were significantly more likely to report low mastery of work. *Goals and choice* accounted for 11 percent of the variance, representing a unique significant contribution (*t* = 6.24, *p* < 0.001). *Involvement* contributed significantly to predict the clinicians’ *mastery of work* scores (*t* = −2.99, *p* < 0.01), and accounted for 3 percent of the variance. Clinicians who perceived that the treatment programmes at their respective facilities promoted inpatient involvement were significantly more likely to experience lower levels of mastery of work. Finally, as in the first regression analysis, the *individually tailored and varied* subscale did not contribute significantly to predicting clinicians’ *mastery of work* scores (*t* = 1.09, *p* = 0.28).

## 4. Discussion

This study investigated the role of recovery orientation in clinicians’ perceptions of various factors that have been associated with work-related satisfaction in inpatient SUD treatment. Clinicians’ perceptions of recovery orientation at treatment facilities were investigated using the following three RSA-N subscales: *goals and choice*, *involvement,* and *individually tailored and varied*. Clinicians’ work-related satisfaction was assessed using the following two scales from QPS_Nordic_: *positive challenges at work* and *mastery of work*. Two block-wise multiple regression analyses were performed. *Positive challenges at work* was used as the hypothesised outcome variable in the first model, and *mastery of work* was used in the second model.

The results suggest that clinicians’ work-related satisfaction was positively influenced by inpatients’ opportunities to pursue their goals and choices, and negatively influenced by the degree of inpatient involvement at the facilities. Characteristics of the treatment practice, such as personalised measures or diversity of treatment options, did not significantly affect work-related satisfaction.

The results also show that the *goals and choice* subscale had the strongest influence on clinicians’ reports on both the outcome variables in the regression analyses. This subscale provides information on the degree to which clinicians perceive that measures at their respective treatment facilities promote inpatients’ individually defined goals, and the extent to which inpatients’ choices are supported and respected. Clinicians who perceived that these choices were respected, and that the practice at their respective treatment facilities provided inpatients with the opportunity to define and pursue their goals, reported significantly higher levels of mastery of work and positive challenges at work.

Previous studies have explored how recovery orientation, as a unidimensional variable, contributes to work-related satisfaction, without exploring the influence of individual recovery-orientated interventions. Kraus and Stein [12] reported that mental health clinicians who perceive the services in which they work as recovery-oriented, are more likely to experience higher levels of professional accomplishment and job satisfaction than those without such an experience. Osborn and Stein [11] found that the variance observed in mental health providers’ reports of job satisfaction could partly be accounted for by their perceptions of recovery orientation at their respective agencies. Rabenschlag, Konrad, Rueegg and Jaeger [52] showed that clinicians’ perceptions of the practice as being recovery-oriented increased and their job satisfaction improved after implementing recovery orientation in an SUD and mental illness treatment facility. Moreover, one year later, job satisfaction was significantly higher among clinicians working at the facility compared to a control group [52].

Our findings are in line with these studies. Moreover, our study contributes to the literature by exploring the influence of individual domains of recovery orientation on clinicians’ work-related satisfaction. Specially, the results from our study contribute by illustrating that treatment measures that enable inpatients to pursue their goals and choices seem to exert a positive influence on clinicians’ work-related satisfaction. As such measures appear to be beneficial for both inpatients and clinicians, they should be enhanced in the programme at SUD treatment facilities.

The results of both regression analyses in this study showed that clinicians who perceived a higher degree of inpatient involvement at their treatment facilities were more likely to report lower levels of positive challenges at work, and mastery of work. The *involvement* subscale provides information about the extent to which inpatients are involved in the development and planning of the treatment programme and staff training, and whether inpatients are represented in advisory boards of treatment facilities. These results were not as significant, and did not predict as high a variance in the outcome variables as the *goals and choice* subscale. However, the direction of the results of both regression analyses was similar, and the contribution of *involvement* was significant, although it had a stronger influence on *mastery of work* than on *positive challenges at work*.

These results are in line with those of Jorgensen and Rendtorff [75], who suggested that patient involvement can be perceived by mental health professionals as time-consuming, challenging, and sometimes frustrating. The patients become experts when the treatment practice is oriented towards patient involvement and shared decision making. Other studies have also suggested that clinicians may find that patient involvement challenges their expertise as professionals [23,34,76,77]. This may contribute to explaining this study’s findings, suggesting that clinicians’ perceptions of mastery of work seemed to be influenced by inpatient involvement at their facilities.

Additionally, the results of this study showed that *quantitative job demands* also contributed significantly to clinicians’ perceptions of mastery of work. High workloads, working overtime, and low work-related self-efficacy among clinicians have been associated with low satisfaction, burnout, and impaired well-being at work [4,6,8]. This may partly explain the negative influence that *involvement* seemed to have on clinicians’ perceptions of mastery at work.

Some measures have been suggested as beneficial for promoting user involvement in services for people with SUD or mental health problems. Clinicians need adequate information about the subject of user involvement and the measures used to achieve it [78,79]. Moreover, they need sufficient time and resources to implement these measures [75]. Such measures may reduce the pressure on clinicians and increase the information provided to inpatients about their opportunities for involvement.

Finally, our results showed that variance in either *positive challenges at work* or *mastery of work* scores could not be accounted for by clinicians’ perceptions of treatment measures as individually tailored and varied. The *individually tailored and varied* subscale concerns characteristics of the treatment programme, and provides information on the extent to which clinicians believe that their treatment facilities offer a diversity of treatment options that can be customised to meet inpatients’ individual needs. Recovery orientation shares certain values with patient-centred and trauma-informed care, such as empowering and individualised treatment measures [80,81,82]. The results of this study diverge from previous findings regarding the influence of trauma-informed and patient-centred care on clinicians’ work-related satisfaction. In a synthesis of several systematic reviews, Park, Giap, Lee, Jeong, Jeong and Go [57] found that a patient-centred orientation at the workplace has a positive influence on clinicians’ job satisfaction and confidence in their work. Hales, Green, Bissonette, Warden, Diebold, Koury and Nochajski [56] reported that clinicians’ workplace satisfaction, associated with fulfilment and possessing necessary skills, improved after trauma-informed care was implemented at an SUD treatment facility.

Park, Giap, Lee, Jeong, Jeong and Go [57] and Hales, Green, Bissonette, Warden, Diebold, Koury and Nochajski [56] found a positive association between clinicians’ work-related satisfaction and empowering treatment measures, such as promoting inpatients’ goals and choices. Our results are partly consistent with these findings. Conversely, unlike these studies, we did not find an association between individualised treatment measures, such as providing individually tailored services, and clinicians’ work-related satisfaction.

These conflicting results may be attributed to several factors, one being that such therapeutic orientations are not directly comparable. However, the value of providing treatment measures based on patients’ individual needs is clearly present in recovery-oriented, patient-centred and trauma-informed care alike [80,83]. Our findings may indicate that variance in clinicians’ work-related satisfaction should not be attributed to perceptions of treatment measures as individually tailored and varied. Further research is required to identify confounding factors, and to gain a more profound understanding of the role of the individual domains of recovery orientation on clinicians’ work-related satisfaction.

### Limitations

Certain limitations of this study should be noted. First, the questionnaire did not gather information on education level, income, and employment status (full- or part-time). One reason for this was that we wished to limit the number of items in the questionnaire, due to the workload and time pressure that clinicians in the SUD field experience [5,6]. Another reason is that research suggests that the variance in clinicians’ work-related satisfaction cannot be explained by income, education level, or employment status [5,11,12]. Second, the study’s cross-sectional design means that no causality can be established, and that confounding factors may be present. This should be considered when interpreting the results of this study. However, the hypothesised directions of influence between the variables were based on previous findings [11,12,52]. Third, the data were obtained using a self-report questionnaire, which entails the risk of social desirability bias. However, the risk was reduced by the fact that the questionnaires were completed anonymously. Lastly, the response rate, albeit acceptable, was moderate (46 percent). However, the sample included participants from 50 of 54 eligible inpatient SUD treatment facilities in Norway, which suggests that it is broadly representative of the target population.

## 5. Conclusions

This study’s main findings suggest that clinicians’ work-related satisfaction is partly influenced by the contextual psychosocial factors at play in a recovery-oriented treatment framework. Work-related satisfaction is positively influenced by inpatients’ opportunities to pursue their goals and choices, and negatively influenced by inpatient involvement. The characteristics of the treatment practice, such as personalised measures or diversity of treatment options, do not significantly affect work-related satisfaction. Treatment measures that enable inpatients to pursue their goals and choices seem to exert a positive influence on clinicians’ work-related satisfaction. Such measures should be enhanced, as they appear to be beneficial for both inpatients and clinicians in SUD treatment facilities. Furthermore, to overcome the barriers that are associated with the promotion of user involvement in the SUD and mental health fields, clinicians should be educated on the subject and content of inpatient involvement. Clinicians should also be supported and involved in the process of implementing measures to increase inpatient involvement. This study’s findings are in line with previous research showing that recovery orientation has a positive influence on clinicians’ work-related satisfaction. Although further research is needed to confirm them, these findings indicate that a recovery-oriented framework is beneficial for both inpatients and clinicians.

## Figures and Tables

**Table 1:** Participants’ personal and professional characteristics (*n* = 407).

Variable	*n* (%)	Mean (SD)
Age (years)		44.7 (10.6)
Female	275 (68)	
Experience in the SUD field (years)		9.96 (7.38)
Experience in SUD treatment (years)		7.70 (6.45)
**Job title/Quantitative job demands (1–5)**		2.9 (0.73)
Medical staff	145 (35)	3 (0.77)
Social worker	64 (16)	2.9 (0.66)
Psychologist or therapist	44 (11)	3.1 (0.55)
Other staff	154 (38)	2.83 (0.75)

SUD = Substance use disorder.

**Table 2:** Multiple block-wise regression analysis of positive challenges at work.

Block	Predictor	B	β	t	Adjusted R²	ΔR²
1	Age	0.01	0.25	4.41 ***	0.06	
2	Age	0.01	0.25	4.56 ***		
	Goals and choice	0.50	0.35	6.44 ***	0.18	0.12 ***
3	Age	0.01	0.24	4.45 ***		
	Goals and choice	0.60	0.41	6.60 ***		
	Involvement	−0.09	−0.13	−2.06 *	0.19	0.01 *
4	Age	0.01	0.24	4.31 ***		
	Goals and choice	0.58	0.40	5.59 ***		
	Involvement	−0.09	−0.14	−2.01 *		
	Individually tailored and varied	0.02	0.03	0.33	0.18	–

The demographic variables controlled for in the preliminary analysis were gender, *quantitative job demands*, experience in the SUD field, experience in inpatient SUD treatment and job title. * *p* < 0.05, *** *p* < 0.001.

**Table 3:** Multiple block-wise regression of mastery of work.

Block	Predictor	B	β	t	Adjusted R²	ΔR²
1	Quantitative job demands	−0.14	−0.23	−3.95 ***	0.05	
2	Quantitative job demands	−0.13	−0.20	−3.67 ***		
	Goals and choice	0.39	0.34	6.24 ***	0.16	0.11 ***
3	Quantitative job demands	−0.13	−0.20	−3.74 ***		
	Goals and choice	0.50	0.44	6.96 ***		
	Involvement	−0.10	−0.19	−2.99 ***	0.18	0.03 ***
4	Quantitative job demands	−0.12	−0.19	−3.51 ***		
	Goals and choice	0.46	0.40	5.56 ***		
	Involvement	−0.12	−0.22	−3.17 ***		
	Individually tailored and varied	0.06	0.08	1.09	0.18	<0.01

The demographic variables controlled for in preliminary analysis were gender, age, experience in the SUD field, experience in inpatient SUD treatment and job title. *** *p* < 0.001.

## Data Availability

The datasets generated and/or analysed during the current study are not publicly available due to ethical restrictions but are available from the corresponding author on reasonable request.
