# Erratum: Johannessen et al. Work-Related Satisfaction among Clinicians Working at Inpatient Treatment Facilities for Substance Use Disorder: The Role of Recovery Orientation. *Int. J. Environ. Res. Public Health* 2021, *18*, 7423

**DOI:** 10.3390/ijerph182111461

**Published:** 2021-10-31

**Authors:** Dagny Adriaenssen Johannessen, Trond Nordfjærn, Amy Østertun Geirdal

**Affiliations:** 1 Blue Cross East, 0182 Oslo, Norway; 2 Department of Social Work, Child Welfare and Social Policy, OsloMet—Oslo Metropolitan University, 0130 Oslo, Norway; amyoge@oslomet.no; 3 Department of Psychology, Norwegian University of Science and Technology (NTNU), 7491 Trondheim, Norway; trond.nordfjarn@ntnu.no; 4 Department of Research and Development, Clinic of Substance Use and Addiction Medicine, St. Olavs University Hospital, 7006 Trondheim, Norway

## Additional Affiliation(s)

In the published article [1], there was an error regarding the affiliations for **Trond Nordfjærn**. In addition to the affiliation **Department of Psychology, Norwegian University of Science and Technology (NTNU), 7491 Trondheim, Norway** should be **Department of Research and Development, Clinic of Substance Use and Addiction Medicine, St. Olavs University Hospital, 7006 Trondheim, Norway**. The authors apologize for any inconvenience caused and state that the scientific conclusions are unaffected. The original article has been updated.
